# Postvaccination SARS-CoV-2 Infections Among Skilled Nursing Facility Residents and Staff Members — Chicago, Illinois, December 2020–March 2021

**DOI:** 10.15585/mmwr.mm7017e1

**Published:** 2021-04-30

**Authors:** Richard A. Teran, Kelly A. Walblay, Elizabeth L. Shane, Shannon Xydis, Stephanie Gretsch, Alexandra Gagner, Usha Samala, Hyeree Choi, Christy Zelinski, Stephanie R. Black

**Affiliations:** ^1^Epidemic Intelligence Service, CDC; ^2^Communicable Disease Program, Chicago Department of Public Health, Illinois; ^3^Immunization Program, Chicago Department of Public Health, Illinois.

Early studies suggest that COVID-19 vaccines protect against severe illness (*1**)*; however, postvaccination SARS-CoV-2 infections (i.e., breakthrough infections) can occur because COVID-19 vaccines do not offer 100% protection (*2*,*3*). Data evaluating the occurrence of breakthrough infections and impact of vaccination in decreasing transmission in congregate settings are limited. Skilled nursing facility (SNF) residents and staff members have been disproportionately affected by SARS-CoV-2, the virus that causes COVID-19 (*4*,*5*), and were prioritized for COVID-19 vaccination (*6*,*7*). Starting December 28, 2020, all 78 Chicago-based SNFs began COVID-19 vaccination clinics over several weeks through the federal Pharmacy Partnership for Long-Term Care Program (PPP).^†^ In February 2021, through routine screening, the Chicago Department of Public Health (CDPH) identified a SARS-CoV-2 infection in a SNF resident >14 days after receipt of the second dose of a two-dose COVID-19 vaccination series. SARS-CoV-2 cases, vaccination status, and possible vaccine breakthrough infections were identified by matching facility reports with state case and vaccination registries. Among 627 persons with SARS-CoV-2 infection across 75 SNFs since vaccination clinics began, 22 SARS-CoV-2 infections were identified among 12 residents and 10 staff members across 15 facilities ≥14 days after receiving their second vaccine dose (i.e., breakthrough infections in fully vaccinated persons). Nearly two thirds (14 of 22; 64%) of persons with breakthrough infections were asymptomatic; two residents were hospitalized because of COVID-19, and one died. No facility-associated secondary transmission occurred. Although few SARS-CoV-2 infections in fully vaccinated persons were observed, these cases demonstrate the need for SNFs to follow recommended routine infection prevention and control practices and promote high vaccination coverage among SNF residents and staff members.

CDPH monitors SNF SARS-CoV-2 infections using a data triangulation method that matches the SARS-CoV-2 test results from nucleic acid amplification tests (NAATs, such as reverse transcription–polymerase chain reaction [RT-PCR]) and antigen tests reported to the Illinois’ National Electronic Disease Surveillance System with facility-reported line lists of SARS-CoV-2 test results from routine screening testing.^§^ In February 2021, CDPH began matching records to Illinois’ Comprehensive Automated Immunization Registry Exchange to identify breakthrough infections. After identifying SARS-CoV-2 infection in a SNF resident 16 days after receipt of a second vaccine dose, CDPH initiated an investigation to quantify breakthrough infections across all facilities, evaluate symptoms and clinical outcomes, and assess potential secondary transmission. Vaccine effectiveness was not evaluated.

A facility’s investigation period started on its first vaccination clinic date and ended March 31, 2021.^¶^ A confirmed case of SARS-CoV-2 infection was defined as a positive SARS-CoV-2 NAAT or antigen test result from a respiratory specimen collected from a resident or staff member during the investigation period. Consistent with CDC guidance, a vaccine breakthrough infection in a resident or staff member was defined as a receipt of a positive SARS-CoV-2 NAAT or antigen test result from a respiratory specimen collected ≥14 days after completing the two-dose COVID-19 vaccination series.** Infection prevention specialists conducted case investigations to assess symptoms, clinical outcomes, and close contact information.

SARS-CoV-2 incidence during the investigation period was assessed across four groups based on vaccination status at the time a positive respiratory specimen was collected: 1) unvaccinated (never received a COVID-19 vaccine dose); 2) partially vaccinated (received one dose of a two-dose series); 3) vaccinated but not immune (received two doses of a two-dose series but <14 days had elapsed since the second dose); and 4) fully vaccinated (received two doses of a two-dose series and ≥14 days had elapsed since the second dose). In addition to routine facility follow-up, CDPH actively monitored facilities with breakthrough infections for 28 days to identify whether any new cases occurred in close contacts of the person with breakthrough infection.^††^ Analyses were completed using SAS (version 9.4; SAS Institute). This activity was reviewed by CDC and was conducted consistent with applicable federal law and CDC policy.^§§^

During the investigation period, an estimated 7,931 SNF residents and 6,834 staff members received two doses of COVID-19 vaccine. A total of 627 confirmed SARS-CoV-2 infections were identified across 75 of the 78 Chicago-based SNFs, including 353 (56%) among residents and 274 (44%) among staff members during the investigation period (Table 1). Three facilities had no confirmed SARS-CoV-2 infections after their first vaccination clinic. Approximately one half (47%) of resident cases occurred in men, 42% were in non-Hispanic Black persons, and the median age was 71 years. More than two thirds (72%) of staff member cases were in women, 38% were in non-Hispanic Black persons, and the median age was 42 years. Among the 627 cases, 447 (71%) occurred in unvaccinated persons, 145 (23%) in partially vaccinated persons, 13 (2%) in vaccinated but not immune persons, and 22 (4%) in fully vaccinated persons (Figure). These breakthrough infections occurred in 12 residents and 10 staff members and accounted for 16% (22 of 136) of SNF-associated cases occurring across all facilities ≥14 days after the second vaccination clinic at the respective facilities. No demographic or clinical differences were observed by vaccination status.

**TABLE 1:** Number and percentage of skilled nursing facility residents and staff members with a positive confirmed SARS-CoV-2 test result, by demographic and clinical characteristics and vaccination status — Chicago, Illinois, December 2020–March 2021

Characteristic	Vaccination status of residents and staff members with SARS-CoV-2 infections, no. (column %)				
	Total (n = 627)	Unvaccinated* (n = 447)	Partially vaccinated* (n = 145)	Vaccinated but not immune* (n = 13)	Fully vaccinated with breakthrough infection* (n = 22)
Median age (IQR)	60.0 (43.0–73.0)	57.0 (39.0–71.0)	65.0 (50.0–79.0)	66.0 (58.0–79.0)	61.5 (41.0–73.0)
**Sex**					
Female	376 (60.0)	265 (59.3)	89 (61.4)	7 (53.9)	15 (68.2)
Male	237 (37.8)	168 (37.6)	56 (38.6)	6 (46.2)	7 (31.8)
Unknown	14 (2.2)	14 (3.1)	0 (—)	0 (—)	0 (—)
**Race/Ethnicity**					
Hispanic/Latino	58 (9.3)	37 (8.3)	16 (11.0)	1 (7.7)	4 (18.2)
Asian, non-Hispanic	24 (3.8)	11 (2.5)	9 (6.2)	1 (7.7)	3 (13.6)
Black, non-Hispanic	252 (40.2)	193 (43.2)	44 (30.3)	7 (53.9)	8 (36.4)
White, non-Hispanic	144 (23.0)	79 (17.7)	55 (37.9)	3 (23.1)	7 (31.8)
Other,^†^ non-Hispanic	16 (2.6)	12 (2.7)	4 (2.8)	0 (—)	0 (—)
Unknown	133 (21.2)	115 (25.7)	17 (11.7)	1 (7.7)	0 (—)
**Role**					
Resident	353 (56.3)	235 (52.6)	97 (66.9)	9 (69.2)	12 (54.6)
Staff member	274 (43.7)	212 (47.4)	48 (33.1)	4 (30.8)	10 (45.5)
**Symptoms** **^§^**					
Yes	92 (14.7)	62 (13.9)	21 (14.5)	1 (7.7)	8 (36.4)
No	34 (5.4)	15 (3.4)	5 (3.5)	0 (—)	14 (63.6)
Unknown	501 (79.9)	370 (82.8)	119 (82.1)	12 (92.3)	0 (—)
**Hospitalizations**					
Yes	123 (19.6)	90 (20.1)	27 (18.6)	2 (15.4)	4 (18.2)^¶^
No	504 (80.4)	357 (79.9)	118 (81.4)	11 (84.6)	18 (81.8)
**Deaths**					
Yes	21 (3.4)	14 (3.1)	6 (4.1)	0 (—)	1 (4.6)
No	606 (96.7)	433 (96.9)	139 (95.9)	13 (100.0)	21 (95.5)
**Previous positive SARS-CoV-2 result**					
Yes	41 (6.5)	22 (4.9)	9 (6.2)	4 (30.8)	6 (27.3)
No	586 (93.5)	425 (95.1)	136 (93.8)	9 (69.2)	16 (72.7)

**Abbreviations:** I-NEDSS = Illinois’ National Electronic Disease Surveillance System; IQR = interquartile range.

* Unvaccinated: received no COVID-19 vaccine doses; partially vaccinated: received one dose; vaccinated but not immune: received two doses but <14 days had elapsed since receipt of second dose; and fully vaccinated with breakthrough infection: received two doses and then received a positive SARS-CoV-2 test result ≥14 days after receipt of the second dose.

^†^ Persons with the following races listed in I-NEDSS as American Indian or Alaska Native, Native Hawaiian or other Pacific Islander, other, or multiracial were categorized as non-Hispanic other.

^§^ Data on symptoms were extracted from I-NEDSS for unvaccinated, partially vaccinated, and vaccinated but not immune persons. Most COVID-19 case reports are entered into I-NEDSS through electronic laboratory reporting and provide minimal information (e.g., name, date of birth, and laboratory test and results) needed to meet reporting requirements. Data on symptoms in persons with breakthrough infections were supplemented with details from case investigations, which were not completed for nonbreakthrough cases. Symptom status for many persons with nonbreakthrough infection cases is unknown.

^¶^ Two residents were hospialtalized for COVID-19–related reasons. Two additional residents were hospitalized for non-COVID-19–related reasons.

**FIGURE:**
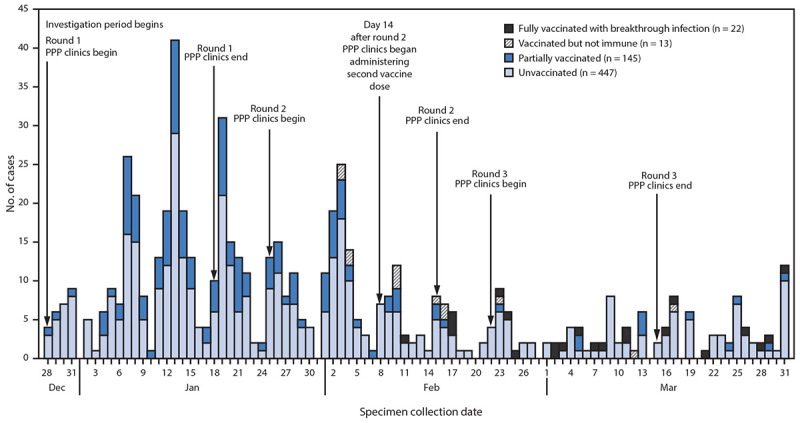
Confirmed SARS-CoV-2 infections (n = 627) among residents and staff members at 75* skilled nursing facilities, by specimen collection date and vaccination status^†^ — Chicago, Illinois, December 2020–March 2021 **Abbreviation:** PPP = Federal Pharmacy Partnership for Long-Term Care Program. * Among 78 Chicago-based facilities. Three facilities had no confirmed SARS-CoV-2 infections. † Unvaccinated: received no COVID-19 vaccine doses; partially vaccinated: received one dose; vaccinated but not immune: received two doses but <14 days had elapsed since receipt of second dose; fully vaccinated with breakthrough infection: received two doses and then received a positive SARS-CoV-2 test result ≥14 days after receipt of the second dose.

Among the 22 breakthrough infections, 18 (82%) were detected in persons who received testing as part of routine screening, and four (18%) occurred in residents who received testing before a hospital admission or procedure. Among the 18 breakthrough infections identified during routine screening, 14 were detected (across 10 facilities) while residents were receiving weekly testing from the facilities; staff members at all facilities were receiving testing at least weekly. The median interval from second dose to collection of a positive SARS-CoV-2 specimen was 29 days (interquartile range [IQR] = 23–42 days). The median interval between most recent positive NAAT result and last known negative test result was 7 days (IQR = 7–14 days). Two-dose vaccination coverage among residents and staff members at facilities with breakthrough infections ranged from 62% to 96% and 18% to 85%, respectively. Among the 15 facilities with breakthrough cases, attack rates^¶¶^ for unvaccinated and vaccinated residents were 15% (89 of 604) and 0.8% (15 of 1,781), respectively. Among staff members, attack rates for unvaccinated and vaccinated persons were 6% (62 of 992) and 1% (12 of 1,135), respectively. Eleven facilities reported a total of 41 confirmed cases within 28 days after initial breakthrough infection at a facility (Table 2).*** No facility-associated secondary transmission was determined to have occurred because the new cases that occurred after the initial breakthrough infection were not close contacts of the persons with breakthrough infections.

**TABLE 2:** Skilled nursing facility residents and staff members with SARS-CoV-2 breakthrough infections,* by facility and clinical characteristics — Chicago, Illinois, December 2020–March 2021

Facility	Patient	Residents and staff members with breakthrough infection										Facilities	
		Sex, age (yrs)	Role	Symptoms	Hospitalized	Death	No. of days between second vaccine dose and positive specimen collection	Ct value^†^	Previous positive SARS-CoV-2 test result	No. of days between initial and most recent positive test result	No. of days between last negative SARS-CoV-2 test result and postvaccination positive test	No. of facility cases occurring after breakthrough infection^§^	No. of cases considered close contacts^¶^
A	1	F, 75	Resident	Pneumonia, seizure	Yes, not COVID-19 related**	No	16	—	No	—	23	3	0
B	2	F, 63	Staff	None	No	No	21	—	No	—	6	3	0
	3	F, 36	Staff	HA, fatigue	No	No	29	—	No	—	7	2	0
C	4	F, 83	Resident	None	No	No	18	26.2	No	—	7	2	0
D	5	M, 64	Resident	None	Yes, not COVID-19 related††	No	15	—	Yes	96	106	2	0
	6	M, 73	Resident	Pneumonia, weakness	Yes, COVID-19 related^§§^	No	21	—	No	—	112	2	0
	7	F, 70	Resident	None	No	No	42	28.8	No	—	7	4	0
E	8	F, 82	Resident	Fatigue, cough	No	No	19	26.9	No	—	29	9	0
	9	F, 95	Resident	None	No	No	28	—	Yes	303	9	5	0
	10	F, 29	Staff	HA	No	No	34	50.0^¶¶^	No	—	7	1	0
F	11	F, 46	Resident	None	No	No	29	—	Yes	112	8	0	NA
G	12	F, 36	Staff	Chills, myalgia, HA, sore throat, fatigue, cough, loss of taste or smell	No	No	27	29.9	No	—	4	2	0
	13	F, 46	Staff	Sore throat, nausea, diarrhea	No	No	51	27.7	No	—	10	2	0
H	14	M, 41	Staff	None	No	No	29	32.6	Yes	214	7	1	0
I	15	F, 37	Staff	None	No	No	39	28.6	No	—	5	1	0
J	16	M, 60	Resident	None	No	No	31	38.5	No	—	7	1	0
	17	M, 26	Staff	None	No	No	53	—	No	—	14	1	0
K	18	F, 57	Staff	None	No	No	27	28.3	No	—	7	7	0
L	19	F, 49	Staff	None	No	No	42	16.9	No	—	7	0	NA
M	20	M, 77	Resident	None	No	No	45	20.5	No	—	220	0	NA
N	21	F, 70	Resident	None	No	No	56	—	Yes	137	7	4	0
O	22	M, 66	Resident	Pneumonia, fatigue, cough, SOB, difficulty breathing	Yes, COVID-19 related***	Yes***	46	—	Yes	152	13	0	NA

**Abbreviations:** Ct = cycle threshold; F = female; HA = headache; M = male; NA = not applicable; NAAT = nucleic acid amplification test; SOB = shortness of breath.

* Receipt of a positive SARS-CoV-2 NAAT (e.g., reverse transcription–polymerase chain reaction) or antigen test result from a respiratory specimen collected ≥14 days after completing the two-dose COVID-19 vaccination series.

^†^ Missing Ct values are for specimens that were discarded by the laboratory and unavailable for sequencing.

^§^ Cases identified within a 28-day monitoring window from the date of specimen collection for the breakthrough infection. For facilities with multiple breakthrough infections, 28-day monitoring windows might overlap, and new facility cases might be listed multiple times.

^¶^ When a new case in a facility was identified, infection prevention specialists determined whether the person with the case met criteria to be considered a close contact of the person with the breakthrough infection. Data in this column represent the number of cases in persons that met the definition of a close contact and occurred after identification of the breakthrough infection as of April 12, 2021.

** Resident A1 had a positive SARS-CoV-2 test result 16 days after receiving the second dose of COVID-19 vaccine, which was an incidental finding when admitted to the hospital for new onset of seizures. The positive result might have been related to an episode of pneumonia diagnosed 9 days after the second dose. COVID-19 testing was not associated with compatible symptoms, and this resident was included because the resident’s case met the laboratory-based breakthrough infection definition. This resident likely did not experience a breakthrough infection because previous pneumonia onset is suggestive of SARS-CoV-2 infection before full immunization.

^††^ Resident D5 had a positive preprocedural SARS-CoV-2 test result and was subsequently hospitalized for non-COVID-19–related reasons, including multiple falls and a bloodstream infection related to a midline catheter.

^§§^ Resident D6 was hospitalized for COVID-19-related reasons because of weakness and loss of appetite in association with pneumonia diagnosed at hospital admission.

^¶¶^ Based on the symptomatic disease case definition outlined by the Council of State and Territorial Epidemiologists, patient E10 had nonspecific COVID-19 symptoms. Previous testing history was examined using the Illinois’ National Electronic Disease Surveillance System. No known previous positive SARS-CoV-2 were identified for this patient.

*** Resident O22 was hospitalized for COVID-19-related reasons because of fatigue, cough, SOB, and difficulty breathing. SARS-CoV-2 test result was positive, and resident received a diagnosis of pneumonia requiring hospital admission and intubation in the intensive care unit for hypoxic respiratory failure. Concomitant infections including group B streptococcal bacteremia and *Pseudomonas* urinary tract infection were also identified. The resident died 7 days after hospital admission.

Among the 22 persons with breakthrough infections, 14 (64%; eight residents and six staff members) were asymptomatic (Table 2). Three symptomatic persons (B3, E10, and G13) had mild, nonspecific symptoms; two (E8 and G12) had mild, specific symptoms; and three (A1, D6, and O22) had diagnosed pneumonia.^†††^ Four residents were hospitalized: two (D6 and O22) for COVID-19–related reasons and two (A1 and D5) for reasons unrelated to COVID-19; one resident (O22) died.

Resident A1 received a diagnosis of pneumonia 9 days after receiving the second COVID-19 vaccine dose and 7 days before receiving a positive SARS-CoV-2 test result (Table 2). Although the timing of the patient’s positive SARS-CoV-2 test result met the definition of a breakthrough infection, the clinical history indicated that the infection likely occurred <14 days after the second dose. Resident D6 was hospitalized for weakness and loss of appetite in association with pneumonia. Resident O22 experienced fatigue and respiratory symptoms and received a diagnosis of pneumonia. This patient had a positive SARS-CoV-2 test result on hospital admission and had concomitant group B *β*-hemolytic streptococcal bacteremia and a *Pseudomonas* urinary tract infection and died 7 days after hospital admission. The death certificate listed complications of COVID-19 infection as primary cause of death; underlying conditions were hypertension, diabetes mellitus, and chronic kidney disease.

Among 12 available specimens from seven patients with breakthrough infections, RT-PCR cycle threshold values were >28, indicating low levels of detectable virus. Six persons with breakthrough infections had a previous positive SARS-CoV-2 test result >90 days before the most recent test, including five persons who had negative test results (range = 1–43 tests) between the positive results (Supplementary Figure, https://stacks.cdc.gov/view/cdc/105130) and at least one negative NAAT result <14 days before the postvaccination positive test result. Five persons were asymptomatic during the second infection. Paired specimens for sequence comparison were unavailable.

## Discussion

Twelve SNF residents and 10 staff members had positive SARS-CoV-2 test results ≥14 days after receiving a second COVID-19 vaccine dose (breakthrough infections). Fourteen (64%) were asymptomatic, available RT-PCR cycle threshold values suggest low viral loads, and no facility-associated secondary transmission was detected. Two residents with breakthrough infections experienced COVID-related hospitalizations, one of whom died because of multiple concurrent infections. Although rare, postvaccination breakthrough infection can occur because COVID-19 vaccines do not offer 100% protection (*2*,*3*). Early studies suggest that COVID-19 vaccines protect against severe illness and might be effective at preventing infection (*1*); however, data on the impact of vaccination on transmission in congregate settings are limited. In addition, some persons whose infections met the case definition of a breakthrough infection might have had persistently positive NAAT results after initial infection; however, most had multiple confirmed negative interim test results. Additional data are needed to differentiate breakthrough infections from sequelae of previous infections and to determine whether persons with breakthrough infections can transmit virus.

The results in this report highlight the importance of COVID-19 vaccination in high-risk congregate settings such as SNFs; most fully vaccinated persons were not infected, did not have COVID-19–like symptoms, and did not have severe illness. Despite the identification of positive NAAT results during the investigation period, breakthrough infections did not lead to secondary transmission at these facilities. 

Expanded testing of residents and staff members in these settings in response to clusters or outbreak investigations is also important, regardless of vaccination status, because these persons might have asymptomatic infections.^§§§^ A previous study found that vaccination has an estimated effectiveness of 63% against SARS-CoV-2 infection among SNF residents >14 days after the first dose through 7 days after the second dose (*8*). Additional studies are needed to assess the impact of full vaccination in SNFs and to understand how vaccination in settings that include older adults, immunocompromised persons, and persons with known history of SARS-CoV-2 infection compares with clinical trial efficacy data. Whether vaccinated asymptomatic persons can transmit SARS-CoV-2 is also unknown; therefore, facilities should continue to require residents to quarantine after close contact with an infected person.^¶¶¶^

Vaccine effectiveness estimates for prevention of SARS-CoV-2 infection and COVID-19 were not calculated because CDPH does not have access to SNF electronic medical records, limiting the ability to obtain individual-level data from facilities on all residents and staff members and to calculate person-time among vaccinated and unvaccinated persons who were not infected. Facilities did not have the capacity to provide line lists and vaccination information for noninfected residents and staff members.

The findings in this report are subject to at least four limitations. First, confirming whether patients with a breakthrough infection and a previous positive SARS-CoV-2 test result had a true reinfection or represented persons with prolonged shedding from previous infection was not possible. Intermittent prolonged SARS-CoV-2 shedding is well described (*9*). In addition to two SARS-CoV-2 tests ≥90 days apart, paired respiratory specimens are needed so that their genetic sequences can be compared.**** Data such as epidemiologic links to confirmed cases and clinical course can provide supporting evidence for reinfection but do not definitively identify reinfection events. Second, vaccination data in this report are limited to Chicago residents and persons vaccinated in Chicago; data were unavailable for staff members who were not Chicago residents and were vaccinated outside Chicago. Third, data entry errors or delayed surveillance reporting might prevent record matching, leading to an underestimate of breakthrough infections. Finally, although some specimens were submitted for genotyping to evaluate possible variant strains, results are pending and not yet available.

SNFs should continue to follow recommended infection prevention and control practices,^††††^ including work restrictions, isolation of persons with confirmed cases, quarantine of residents who have had close contact with persons with confirmed cases, routine and outbreak testing of residents and staff members, and use of personal protective equipment, regardless of vaccination status. Maintaining high vaccination coverage among residents and staff members is also important to reduce opportunities for transmission within facilities and exposure among persons who might not have achieved protective immunity after vaccination.

**Summary**

**What is already known about this topic?**

Residents and staff members of skilled nursing facilities (SNFs) are recommended to receive COVID-19 vaccine as a priority group.

**What is added by this report?**

Twenty-two possible breakthrough SARS-CoV-2 infections occurred among fully vaccinated persons ≥14 days after their second dose of COVID-19 vaccine. Two thirds of persons were asymptomatic. A minority of persons with breakthrough infection experienced mild to moderate COVID-19–like symptoms; two COVID-19–related hospitalizations and one death occurred. No facility-associated secondary transmission was identified.

**What are the implications for public health practice?**

SNFs should prioritize vaccination and follow recommended COVID-19 infection prevention and control practices, including following work restrictions, isolation, quarantine, testing of residents and staff members, and use of personal protective equipment.

## Footnotes

* These authors contributed equally to this report.

† A public-private partnership among CDC; CVS Pharmacy; Managed Health Care Associates, Inc.; and Walgreens to provide on-site COVID-19 vaccination of residents and staff members at enrolled long-term care facilities, including SNFs. https://www.cdc.gov/vaccines/covid-19/long-term-care/pharmacy-partnerships.html

§ CDPH advises facilities to routinely test residents at least monthly. Staff members were also required to receive testing routinely, with frequency determined based on community positivity rate and other metrics of interest. Staff members at all Chicago-based SNFs were tested at least twice weekly (until February 4, 2021), then weekly (during February 4–March 18, 2021), then biweekly (from March 18, 2021 through the end of investigation period). In response to a facility outbreak (i.e., a resident or staff member with a case within the past 14 days), all staff members and residents (excluding those who received a positive SARS-CoV-2 test <90 days previously) are required to receive testing at least every 3–7 days until no new cases occur for at least 14 days. At the time a breakthrough infection was identified, frequency of resident testing at the 15 SNFs ranged from monthly to twice per week; frequency of staff member testing ranged from weekly to twice weekly.

¶ First round vaccination clinics occurred during December 28, 2020–January 18, 2021. Persons were included in the study if infection occurred between the first clinic date at their respective facility and March 31, 2021. Moderna COVID-19 vaccination was exclusively administered at Chicago-based SNFs through the federal PPP. Six residents and two staff members received Pfizer-BioNTech COVID-19 vaccine through Chicago-based vaccine providers not participating in the federal PPP or facilities outside the city of Chicago.

** CDC breakthrough infection guidance: https://www.cdc.gov/vaccines/covid-19/health-departments/breakthrough-cases.html. Residents and staff members were excluded from the investigation if they had received 1) a positive SARS-CoV-2 test result from December 28 through the date of their facility’s first vaccination clinic or 2) a known initial positive SARS-CoV-2 test result <90 days before their most recent SARS-CoV-2 test result.

†† For residents, close contact was defined as being within 6 ft of a person for ≥15 minutes over a 24-hour period, regardless of personal protective equipment used. For staff members, close contact was defined as being within 6 ft of a person for ≥15 minutes over a 24-hour period when one or both persons were unmasked.

§§ 45 C.F.R. part 46, 21 C.F.R. part 56; 42 U.S.C. Sect. 241(d); 5 U.S.C. Sect.552a; 44 U.S.C. Sect.3501 et seq.

¶¶ To calculate attack rates, residents and staff members who had never received COVID-19 vaccine (i.e., unvaccinated persons) and those who had received one COVID-19 vaccine dose of a two-dose series (i.e., partially vaccinated persons) were categorized as unvaccinated. Residents and staff members who had received two COVID-19 vaccine doses of a two-dose series and <14 days or ≥14 days had elapsed (i.e., vaccinated but not immune and fully vaccinated persons, respectively) were categorized as vaccinated. Aggregate vaccination data were only available for residents and staff members who received two doses of COVID-19 vaccine and did not have a SARS-CoV-2 infection. In the denominator, differentiation between persons who received two doses <14 days or ≥14 days was not possible.

*** Cases identified within a 28-day monitoring window from the date of specimen collection for the breakthrough infection. For facilities with multiple breakthrough infections, 28-day monitoring windows might overlap, and new facility cases might be listed multiple times. As of April 12, 2021, nine of the 41 cases were breakthrough infections; seven of which are listed (see Table 2) and occurred during the investigation period.

††† Symptoms were categorized as nonspecific, mild, or moderate based on the National Notifiable Diseases Surveillance System and Council of State and Territorial Epidemiologists definition of symptomatic COVID-19. https://wwwn.cdc.gov/nndss/conditions/coronavirus-disease-2019-covid-19/case-definition/2020/08/05/

§§§
https://www.cdc.gov/coronavirus/2019-ncov/hcp/nursing-homes-testing.html

¶¶¶
https://www.cdc.gov/coronavirus/2019-ncov/hcp/infection-control-after-vaccination.html

****
https://www.cdc.gov/coronavirus/2019-ncov/php/invest-criteria.html

††††
https://www.cdc.gov/coronavirus/2019-ncov/hcp/long-term-care.html
